# Epidemic Parotitis

**Published:** 1910-12-31

**Authors:** 


### Diseases of Children.

### EPIDEMIC PAROTITIS.

A more strictly correct designation for mumps would be parotiditis, but the suggestion, which was made long ago, has not been taken up. Mumps is a disease which is now treated with more consideration than was perhaps usual in the past. This is not due to any serious increase either in the frequency or in the severity of the disease, but to a better appreciation and knowledge of its complications. Without these complications the affection would indeed be almost trivial, for speaking generally the mortality of mumps is insignificant, some eighty deaths only being registered annually as due to this cause among the entire population of England and Wales. A very large proportion of cases run their course without any complication, while in young children it is the rule for mumps to be uncomplicated. Nevertheless, this must not excuse the neglect of simple precautions, especially complete rest for a sufficient time and careful watching of the patient.

### Incidence of the Disease.

The occurrence of mumps is most likely between the ages of four and fourteen, though adults are often attacked; but infants are seldom affected. This disease has at times all the characters of an epidemic, but is probably endemic in certain localities, especially in large centres of population. At certain seasons, particularly in the spring and autumn months, the number of cases increases rapidly. The fact that epidemics are more common in cold and damp weather may be connected with the question of the deficient ventilation and the overcrowding associated with such weather.

Epidemics notoriously differ much in type and severity and the frequency of complications, and have sometimes followed on outbreaks of measles. It has been suggested, not without reason, that mouth-breathers are more commonly victims; and it appears that boys exceed in number the affected girls. The incubation period is usually fourteen to twenty-five days, with a majority between the seventeenth and twenty-first days, but extremes have been recorded as early as the fourth day and as late as the thirtieth day after exposure.

Quarantine may well be extended to four weeks, but it is regarded as unnecessary to isolate contacts during the whole of this time, but only during the latter half. The infectiousness of mumps is greatest just before the swelling of the parotid develops—that is to say, just before the diagnosis is made, and lasts about three weeks, or until ten or twelve days after all signs of parotid enlargement have disappeared.

### Differential Diagnosis.

The diagnosis is not usually difficult, for in the first place the causes of a swelling in that region other than mumps are comparatively very rare; and in the second place, the enlargement on the opposite side soon clinches the diagnosis. Temporary hesitation may be caused by swelling due to enlargement of the upper cervical glands or periostitis of the jaw. It has been noted that while in health the opening of Stenson's duct is difficult to see, in the early stages of mumps this opening may be observed as a bright red projection on the mucous membrane of the cheek, and thus be of assistance in making an early diagnosis. Another sign peculiar to mumps has been recorded by Mirchamp: the application to the tongue of some acid solution, such as vinegar, will excite a painful reflex secretion in the gland about to be affected. This may be obtained some days before any swelling is visible, and even in those rare forms where the disease is manifested by testicular inflammation preceding that in the parotid.

As a rule the onset of the disease is not characterised by its severity; during the three or four days it takes to reach its height there will generally be some malaise and stiffness of the jaws, perhaps pain referred to the ear, and a rise in temperature of one or two degrees; more rarely vomiting and convulsions, or even delirium, are observed. The left gland appears to be rather more often first affected than the right, the opposite side becoming involved in from twenty-four to thirty-six hours later. It is exceptional for one gland only to become enlarged; but the second one may remain quiescent for several days, or occasionally throughout the illness. In some cases dryness of the mouth is marked, but the secretion of the saliva is not very often noticeably diminished, though, no doubt, there is some interference temporarily.

### Complications.

It is the complications of mumps which make this disease so interesting, and often the reverse of inconsequential. It may be said at once that severe complicated cases are almost exclusively above the age of twelve. Complications of the parotid inflammation itself may be summarily dismissed as excessively rare, such as gangrene or suppuration; while accidental infection accounts for cellulitis of the neck and œdema of the glottis.

The most important complication is orchitis. The swelling involves the body of the testicle and not the epididymis, and lasts for about a week. The orchitis occurs round about the eighth day after the swelling of the parotid is noticed, is unilateral, and more frequently affects the left testicle. Rarely it is primary, and then its true significance is apt to be overlooked unless mumps is about. Its importance is due to the fact that atrophy may follow, though resolution is commoner; cases of rapid wasting have occurred, so that the testicle has been left the size of a bean after only a few weeks; fortunately bilateral orchitis is very rare. Every case of mumps in boys over twelve years of age ought to be kept in bed until the tenth day.

On the female side complications such as ovaritis, swelling of the labia, and mastitis may occur; in contrast to the age at which complications in boys are commonly met with, quite young girls may be the subjects of the above, though more than half are over twelve. It is said that bilateral ovaritis is more common than bilateral orchitis; atrophy following in such cases may cause sterility.

SOME RARER SEQUELÆ.

Pancreatitis is an interesting complication in view of the close resemblance of this gland to the parotid. Cases of pancreatitis are probably more frequent than the number recorded suggest, as the affection may be mild and unsuspected. This metastatic pancreatitis appears to begin about the fourth to the sixth day, and lasts a week or less. The chief symptom is epigastric pain with tenderness, while vomiting is a usual accompaniment. Evidences of disturbance of the function of the gland may be found, such as fatty stools and perhaps Cammidge's reaction. It is practically impossible ever to make out any swelling of the pancreas on palpation on account of the tenderness and rigidity which are invariably present. There does not seem to be any evidence of subsequent atrophy.

A serious sequel is permanent and incurable nerve-deafness, which is fortunately unilateral. This may arise suddenly, and sometimes before there is much enlargement of the parotid; but it has occurred as late as the eleventh day. It may be associated with otitis media, or may be caused by an exudation or hæmorrhage into the labyrinth and be accompanied by vertigo tinnitus and vomiting.

A long and formidable list of other complications, which have been observed almost exclusively in adults, includes meningitis, pericarditis, pneumonia, jaundice, splenic enlargement, acute nephritis, endocarditis, and optic neuritis. None of these is common, or presents special features. Nephritis has usually occurred during convalescence, and soon clears up, though a fatal case of uræmia has been recorded. Optic neuritis and retinitis are rare, and usually recovered from. Paralysis of the facial nerve may result from mechanical pressure by the swollen parotid.

It should be borne in mind that occasionally cases are met with in which the parotid is spared, and the brunt of the inflammation falls on the submaxillary and sublingual glands; while, as before mentioned, the testicle may be affected before the parotid.

TREATMENT.

The treatment of mumps does not present many difficulties; the important point is to enjoin complete rest, especially in adults and boys over the age of twelve, who will naturally often object. The local application of heat and a fluid diet, on account of the pain on mastication, will meet the requirements of most of the cases. In these days it should hardly be necessary to emphasise the point that due attention must be paid to the cleanliness of the mouth and an antiseptic mouth-wash be prescribed. The application of two or three leeches will be found to afford great relief where the swelling is very tense and painful; but it is well to place the leeches in such positions that the marks shall not be too prominently noticeable while they last.

